# Comparison of Reactive and Non-Reactive Spark Plasma Sintering Routes for the Fabrication of Monolithic and Composite Ultra High Temperature Ceramics (UHTC) Materials

**DOI:** 10.3390/ma6051566

**Published:** 2013-04-29

**Authors:** Roberto Orrù, Giacomo Cao

**Affiliations:** Dipartimento di Ingegneria Meccanica, Chimica e dei Materiali, Centro Studi sulle Reazioni Autopropaganti (CESRA), Unità di Ricerca del Consorzio Interuniversitario Nazionale per la Scienza e Tecnologia dei Materiali (INSTM), Università degli Studi di Cagliari, Piazza D’Armi, Cagliari 09123, Italy; E-Mail: giacomo.cao@dimcm.unica.it

**Keywords:** spark plasma sintering, self-propagating high-temperature synthesis, mechanical activation, ultra high temperature ceramics, borides, carbides

## Abstract

A wider utilization of ultra high temperature ceramics (UHTC) materials strongly depends on the availability of efficient techniques for their fabrication as dense bodies. Based on recent results reported in the literature, it is possible to state that Spark Plasma Sintering (SPS) technology offers a useful contribution in this direction. Along these lines, the use of two different SPS-based processing routes for the preparation of massive UHTCs is examined in this work. One method, the so-called reactive SPS (R-SPS), consists of the synthesis and densification of the material in a single step. Alternatively, the ceramic powders are first synthesized by Self-propagating High-temperature Synthesis (SHS) and then sintered by SPS. The obtained results evidenced that R-SPS method is preferable for the preparation of dense monolithic products, while the sintering of SHS powders requires relatively milder conditions when considering binary composites. The different kinetic mechanisms involved during R-SPS of the monolithic and composite systems, *i.e.*, combustion-like or gradual solid-diffusion, respectively, provides a possible explanation. An important role is also played by the SHS process, particularly for the preparation of composite powders, since stronger interfaces are established between the ceramic constituents formed *in situ*, thus favoring diffusion processes during the subsequent SPS step.

## 1. Introduction

It is well recognized that Zr-, Hf- and Ta- diborides and carbides based composites, the so-called Ultra High Temperature Ceramics (UHTCs), are suitable in several traditional and innovative application fields due to their high melting temperatures (above 2700 °C) as well as additional attractive chemical, physical and mechanical properties (hardness, electrical and thermal conductivity, inertness, resistance in extreme environments, *etc*.) [1,2,3]. Specifically, such characteristics are particularly beneficial in cutting tools, high temperature crucibles, microelectronics, aerospace industries, *etc*. [1,2]. 

Despite the advantages mentioned above, the diffusion and application of this promising class of ceramics is significantly hindered for two main reasons: the difficulties encountered for the fabrication of dense bodies and the inadequate fracture toughness properties, which may lead to the catastrophic collapse of the material [2]. 

As far as the first aspect is concerned, the low intrinsic sinterability of transition metals borides and carbides can be overcome only when adopting severe sintering temperature and pressure conditions. 

A useful way successfully adopted to make consolidation conditions milder as well as to improve the oxidation resistance of UHTCs is represented by the introduction of suitable sintering aids such as SiC [4,5,6,7,8], Si_3_N_4_ [9], MoSi_2_ [10,11], and TaSi_2_ [11]. 

Less severe consolidation conditions can be also needed if starting from powders with relatively high sintering ability, as compared with products synthesized by conventional routes (furnace, solution methods, *etc*.). For instance, the Self-propagating High-temperature Synthesis (SHS) technique, a well-known combustion synthesis method [12,13,14], can be exploited along this direction. Indeed, with respect to analogous products prepared by alternative methods, relatively higher defect concentration are generated in the obtained powders by the extreme heating and cooling rates (up to 200,000 K/min) accompanying the combustion front propagation, as a consequence of the heat liberated by the exothermic reactions taking place [3,15]. Other motivations, such as finer grain size and the formation of strong interfaces among the different ceramic phases formed *in situ* during the synthesis of composite materials, also account for the advantages observed when the SHS method was adopted [16]. 

Nevertheless, the identification of efficient powder consolidation methods still remains a crucial aspect. In particular, the use of the innovative Spark Plasma Sintering (SPS) technique, wherein the powders to be consolidated and/or the die containing them are crossed by an electric pulsed current [17], is considered highly beneficial with respect to conventional Hot Pressing (HP). While relatively long processing times (on the order of hours) are required by the latter technology, sintering phenomena are strongly accelerated (a few minutes) during SPS by the direct passage of the electric current through the powder compact, which significantly increases heating rates due to the Joule effect. Other mechanisms and phenomena have been also hypothesized to justify the advantages obtained when using SPS [17]. These outcomes hold also true when the reaction synthesis and densification is accomplished in one step by the so-called Reactive SPS (R-SPS). 

Based on the consideration above, several dense advanced materials with uniform and fine microstructure are obtained relatively faster by SPS. In particular, various monolithic and composite UHTC products in bulk form have been prepared in the last decade using such rapid consolidation method, as reported in Table 1. Although most of the ceramics listed in this table have been fabricated starting from previously synthesized UHTC powders, products obtained following the R-SPS approach are also reported [8,18,19,20,21].

**Table 1:** Monolithic and composite bulk ultra high temperature ceramics (UHTC) systems investigated by Spark Plasma Sintering (SPS).

System type	Composition	References
Monolithic	ZrB_2_	[21,22,23,24]
	HfB_2_	[8,11,18]
	TaB_2_	[20]
	ZrC	[25]
	TaC	[26,27,28,29]
Binary	ZrB_2_–SiC	[19,30,31,32,33,34,35,36,37]
	ZrB_2_–MoSi_2_	[10]
	ZrB_2_–ZrC	[38,39,40]
	ZrB_2_–CNT	[37]
	ZrB_2_–graphene	[41]
	ZrC–SiC	[42,43]
	ZrC–MoSi_2_	[44]
	ZrC–TiC	[45]
	HfB_2_–SiC	[8,46,47,48]
	HfB_2_–MoSi_2_	[49]
	HfB_2_–TaSi_2_	[11]
	HfC–MoSi_2_	[50]
	TaB_2_–SiC	[51]
	TaC–TaB_2_	[52]
	TaC–SiC	[53]
Ternary	ZrB_2_–ZrC–SiC	[16,54,55,56,57]
	HfB_2_–HfC–SiC	[48]
	TaB_2_–TaC–SiC	[58]

**Figure 1:**
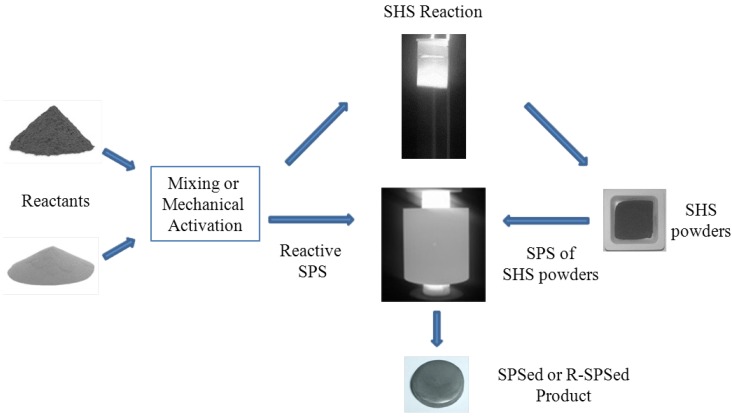
Spark Plasma Sintering routes adopted in this work for the fabrication of bulk ultra high temperature ceramics (UHTCs).

Recent results obtained when the classical and the reactive SPS methods were used for the fabrication of different MB_2_ and MB_2_–SiC (M = Zr, Hf, Ta) dense materials are examined and compared in the present work. Specifically, when the classical processing route is adopted (Figure 1), ceramic powders are first synthesized by SHS starting from Zr/Hf/Ta, B_4_C, and Si reactants and the obtained products are subsequently sintered by SPS. Alternatively, synthesis and consolidation are performed in a single stage using the same reagents mentioned previously. When the low exothermic Ta-based systems are processed, a preliminary mechanical treatment by ball milling of the mixtures is foreseen to activate initial reactants. Particular attention will be focused on the kinetic mechanisms involved during R-SPS of monolithic and composite systems.

## 2. Experimental Section 

Commercial Hf (particle size < 44 μm, >99.6 purity, Alfa-Aesar), Zr (particle size < 44 μm, >98.5 purity, Alfa-Aesar), Ta (particle size < 44 μm, 99.9 purity, Alfa-Aesar), B (amorphous, particle size <9 μm, 95%–97% purity, Fluka), B_4_C (particle size ≈ 1–7 μm, >99.4 purity, Alfa-Aesar), and Si (particle size < 44 μm, >99 purity, Aldrich) powders were employed as starting materials. The processing procedure includes the steps summarized in Figure 1. Specifically, reactants mixing or their mechanical activation by ball milling, for the case of Ta-based systems, were carried out according to the stoichiometries of Reactions (1) and (2) reported in Section 3.

Powders mixing was performed in a SPEX 8000 (SPEX CertiPrep, USA) shaker mill for 30 min using plastic vials and alumina balls. Mechano-chemical activation of Ta-based mixtures was carried out using the same mill apparatus with two steel balls (13 mm diameter, 8 g weight) for 20 min milling time interval and ball to powders or charge ratio (CR) equal to 1.

Cylindrical pellets with a diameter of 10 mm, height of 20–30 mm and green density varying in the range of 50%–62% of the theoretical values were prepared by uni-axially pressing 8–15 g of blended or mechanically activated powders. Detailed information of the set-up used in this work for typical SHS experiments can be found elsewhere [14]. Before sintering, about 4 g of the obtained SHS porous product were ground for 20 min using the milling device mentioned above which is equipped with stainless steel vial with two steel balls (13 mm diameter, 8 g weight).

Both classical and R-SPS experiments (Figure 1) were performed using a Spark Plasma Sintering apparatus (SPS 515 Sumitomo Coal Mining Co Ltd., Japan) under vacuum (20 Pa) conditions. This equipment is able to combine a uniaxial press (max 50 kN) with a DC pulsed current generator (10 V, 1500 A, 300 Hz), thus simultaneously providing an electric current through the processing powders (3–6 g) and/or the graphite die containing them, together with a mechanical load through the die punches. 

Temperature, applied current, voltage, mechanical load and the vertical displacement of the lower electrode were recorded in real time during the process. In particular, temperatures were measured by a C-type thermocouple (Omega Engineering Inc., USA), which was inserted inside a small hole in one side of the graphite die, and/or using the two-color pyrometer indicated above. It is important to note that the measured displacement can be roughly regarded as the degree of powdered compact densification, although thermal expansion of the sample, electrodes, graphite blocks, spacers and plungers, is also responsible for the variation of this parameter. Dense cylindrical specimens with diameter of about 15 mm were typically prepared. For the sake of reproducibility, each experiment was repeated at least twice. Additional details related to SPS experiments are reported elsewhere [19].

The relative densities of dense products were determined by the Archimedes’ method. The theoretical density of the ZrB_2_–SiC, HfB_2_–SiC, and TaB_2_–SiC composite systems, *i.e.*, 5.37, 9.17, and 9.98 g/cm^3^, respectively, were calculated through a rule of mixture [59], by considering the density values of ZrB_2_, HfB_2_, TaB_2_, and SiC equal to 6.1, 11.18, 12.6, 6.4, 12.69, 14.48, and 3.2 g/cm^3^, respectively.

## 3. Results and Discussion

SHS and reactive SPS experiments are performed according to the following reaction stoichiometries:

M + 2(1 + *x*) B → MB_2_ (1)

2M + B_4_C + Si → 2MB_2_ + SiC (2)

where M = Zr, Hf and Ta. The use of an excess of boron (*x* = 0.1) in Reaction (1) is required for the preparation of the diboride product to compensate the partial loss of this reactant during the occurrence of the synthesis reaction. Correspondingly, ZrB_2_, HfB_2_, TaB_2_, ZrB_2_–25 vol % SiC, HfB_2_–26 vol % SiC, and TaB_2_–27.9 vol % SiC, respectively, are the expected ceramics formed from the complete conversion of the initial reactants. 

### 3.1. Monolithic Systems 

#### 3.1.1. HfB_2_

The synthesis Reaction (1) displayed a self-propagating character upon ignition when M = Hf, this fact being a direct consequence of the high enthalpy for the formation of HfB2 from elemental reactants ($-\Delta H_{r}^{o}$ = 335.975 kJ/mol) [60]. In particular, the measured average reaction front velocity and maximum combustion temperature were about 8 mm/s and 2700 °C, respectively.

As mentioned previously, the use of some excess of boron (*x* = 0.1) allows us to synthesize a single phase hexagonal HfB_2_ product, whereas residual Hf was present in the end material synthesized when starting from stoichiometric reactants (*x* = 0). This fact is explained by considering boron loss during synthesis occurrence due to the formation of volatile B_2_O_3_ as a product of B reaction with adsorbed oxygen/moisture present in the original powders. 

After comminuting by ball milling the porous sample, the resulting powders were sintered by SPS under the following conditions. A die with an external diameter equal to 30 mm was used. The electric current *I* was increased from 0 to 1350 A in 10 min, the maximum current level was maintained for additional 20 min (total time *t*_T_ = 30 min), and the applied pressure was kept at 50 MPa. Correspondingly, the maximum temperature measured was in the range 1850–1900 °C and the density of the end-sample was only about 93% of the theoretical value. In principle, the temperature (current) level or the applied load should be increased to improve the density value of the sintered compact. However, it should be noted that, although the maximum current intensity allowable by the SPS apparatus adopted in the present work is 1500 A, the use of electric currents higher than 1350 A was not convenient for various reasons (safety, die/plungers breakage, *etc*.). An increase of the applied pressure or a decrease of the external diameter of the die, while maintaining the sample size unchanged, were both not considered for the same motivation. Therefore, under the experimental conditions adopted in the present work, the fabrication by SPS of completely dense HfB_2_ material was not possible when starting from SHS powders.

**Figure 2:**
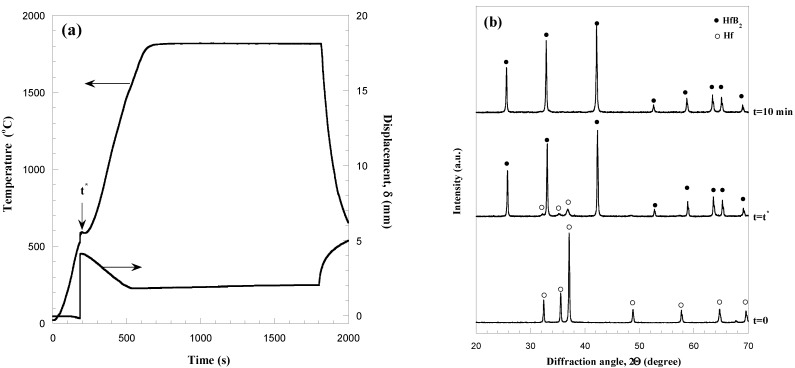
Temperature and displacement time profiles (**a**) measured during reactive SPS of HfB_2_ and (**b**) related compositional changes. Adapted from [8].

The alternative R-SPS approach, consisting in the simultaneous synthesis and densification of HfB_2_ from Hf and B powders, was attempted to this aim. The corresponding sample shrinkage (*δ*) and temperature temporal profiles recorded during the reactive sintering process are reported in Figure 2a for the case when *I* = 1300 A, *t*_T_ = 30 min, and *P* = 20 MPa. It can be observed that as the current was augmented from 0 to 1300 A in 10 min, the temperature correspondingly undergoes a fast increase. Subsequently, the temperature rises at a slower rate and rapidly approaches to an asymptotic value of about 1800 °C which indicates that the thermal equilibrium condition is achieved. 

Temperature evolution during the process is rather smooth, except in correspondence of about 200 s (t^*^), where an irregular variation was observed in the range of 500–600 °C. In order to correlate the SPS process dynamics with the formation mechanism of the HfB_2_ phase, the reacting system composition was monitored by interrupting the application of the current at different time intervals. The related XRD patterns are reported in Figure 2b along with that of the starting mixture. Only original reactants are present if *t* < t^*^. However, the situation completely changes when the abrupt variation of *δ* occurs. Correspondingly, it is clearly seen that elemental reactants are mostly converted to HfB_2_, while a minor amount of unreacted Hf disappears as the SPS process proceeds, thus leading to a single phase material in 10 min sintering time. 

Based on these results, it is then possible to state that the rapid change of *δ* corresponds to the formation of the boride phase through a combustion synthesis mechanism. This statement is supported by the previously mentioned slight increase of the die temperature (Figure 2a) due to the heat released by the exothermic process taking place. Moreover, the displacement decrease occurring after t^*^ is a manifestation of consequent thermal expansion of the sample undergoing sintering, whose effect overcomes powders densification during this stage. Fast reaction and related abrupt sample shrinkage are phenomena observed also in other reacting systems (MoSi_2_, TiC–TiB_2_, *etc*.) processed by R-SPS [17].

**Figure 3:**
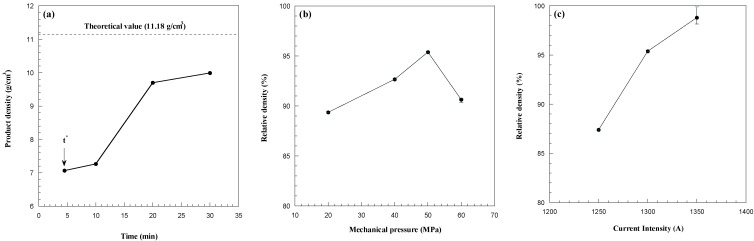
Effect of (**a**) the sintering time (*I* = 1300 A, *P* = 20 MPa); (**b**) the mechanical pressure (*I* = 1300 A, *t*_T_ = 30 min) and (**c**) the applied current (*P* = 50 MPa, *t*_T_ = 30 min) on the density of HfB_2_ samples obtained by R-SPS. Adapted from [8].

The influence of the sintering time, applied mechanical pressure and electric current, on sample density is systematically investigated and the obtained results are reported in Figure 3a–c, respectively. A highly porous product is obtained (*cf.* Figure 3a) immediately after the occurrence of the combustion synthesis reaction (t^*^), while powder consolidation is significantly enhanced during the progress of the SPS process, thus obtaining a 90% dense sample after 30 min total sintering time. To further reduce residual porosity, a two steps mechanical load cycle, where the mechanical pressure is increased immediately after t^*^, was implemented during R-SPS. The corresponding results are reported in Figure 3b for the case when *I* = 1300 A and *t*_T_ = 30 min. 

It is seen that, as the mechanical pressure was augmented from 20 to 50 MPa, the densification level was improved (95.4%), while a further increase (60 MPa) of this parameter gave rise to relatively less dense products. This behavior may be due to the volatilization of some impurities coming from the starting powders and/or formed during synthesis reaction. Indeed, when moderate mechanical loads are applied, the produced gases are allowed to escape from the sample undergoing sintering, whereas they are entrapped in the bulk product if the applied pressure exceeds a certain level, *i.e.*, 50 MPa in the present case. Regarding the choice to increase the applied pressure only after the occurrence of the sharp displacement, it is important to note that the continuous action of high loads could easily lead to dies/plungers breakage during the sudden combustion synthesis event. 

The dependence of sample density on current intensity was then investigated in the range of 1250–1350 A, when setting *P* = 50 MPa. As shown in Figure 3c, as the applied current was augmented from 1250 to 1350 A, and the thermal levels correspondingly increase from about 1750 to 1820 °C, a nearly full dense material (about 99% average relative density) is obtained.

The results described above indicate that the reactive SPS approach is preferable to produce highly dense HfB_2_ with respect to the spark plasma sintering of previously synthesized powders. In this regard, it is worth noting that the consolidation conditions adopted when comparing the two methods are the same, with the only exception of the applied load. Indeed, for the case of the R-SPS route, the mechanical pressure of 50 MPa was applied only after the combustion synthesis reaction, while this value was maintained constant for the entire duration of the SPS process when using SHS powders.

#### 3.1.2. TaB_2_

**Figure 4:**
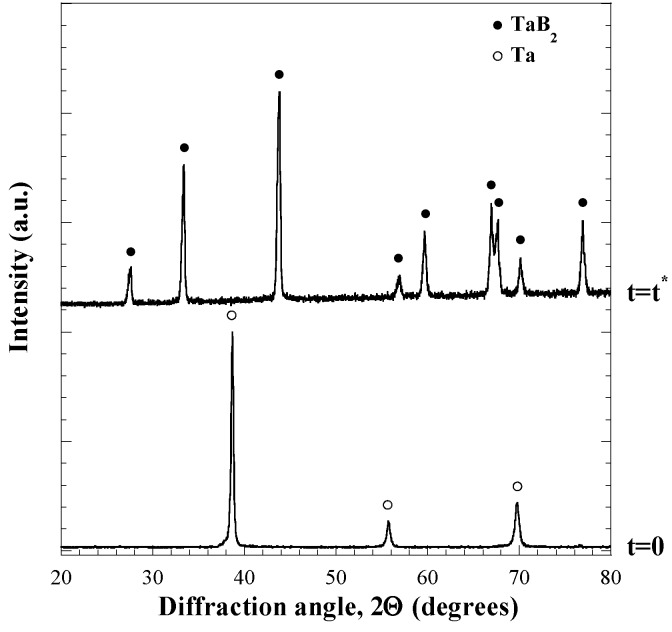
XRD patterns of starting reactants and product obtained immediately after the sharp sample displacement (t^*^) during the R-SPS of TaB_2_. Adapted from [20].

According to the results obtained when investigating the fabrication of monolithic HfB_2_, the reactive sintering processing route was also chosen for the preparation of Ta diboride. A qualitatively similar process dynamic behavior to that described in the previous section and reported in Figure 2 was also observed in this case, so that analogous consideration can be made. Specifically, when the R-SPS process was performed at *I* = 1300 A, *t*_T_ = 30 min, and *P* = 20 MPa, a combustion synthesis event, accompanied by a rapid sample displacement, was observed to occur after about 4 min (t^*^) from the beginning of the electric current application. In addition, as shown in Figure 4, where the XRD pattern of the sample obtained at t^*^ is compared to that of the starting powders, the desired product composition is correspondingly obtained. 

All the phenomena associated to the reactive event, *i.e.*, sharp sample shrinkage, local temperature increase, the displacement decrease after t^*^ as a manifestation of compact expansion, *etc*., are due to the exothermic character ($-\Delta H_{r}^{o}$ = 209.200 kJ/mol) of Reaction (1) with M = Ta [60].

**Figure 5:**
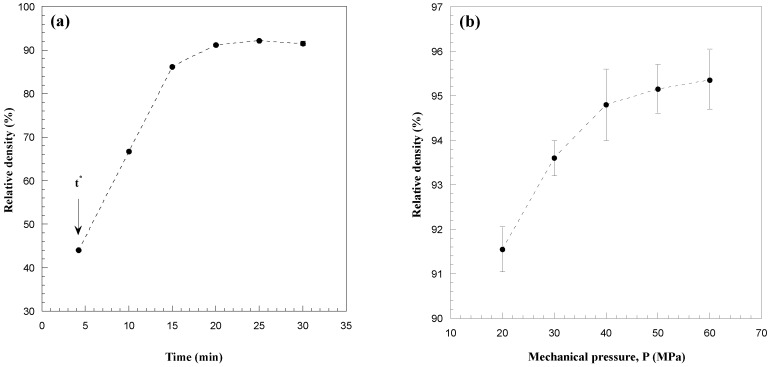
Effect of (**a**) sintering time (*I* = 1300 A, *P* = 20 MPa) and (**b**) mechanical pressure (*I* = 1300 A, *t*_T_ = 30 min) on the density of TaB_2_ samples obtained by R-SPS. Adapted from [20].

The effect of the sintering time on the density of TaB_2_ is displayed in Figure 5a for the case when *P* = 20 MPa. It can be seen that product consolidation is markedly enhanced up to 20 min to achieve about 90% dense samples, while a further increase of the sintering time did not result in significant density changes. With the aim of improving the densification of the boride product, the two steps mechanical load cycle successfully adopted during R-SPS of HfB_2_ was also implemented when processing this system at *I* = 1300 A and *t*_T_ = 30 min. Sample density values obtained when the pressure level was increased from 20 to 60 MPa are plotted in Figure 5b. An improvement in powder compact consolidation to 95%–96% of the theoretical value is correspondingly observed. 

Thus, also the results obtained when considering the TaB_2_ system confirm that the reactive processing method is effective and promising for the fabrication of massive monolithic UHTC materials.

### 3.2. Binary Systems 

#### 3.2.1. ZrB_2_–SiC

**Figure 6:**
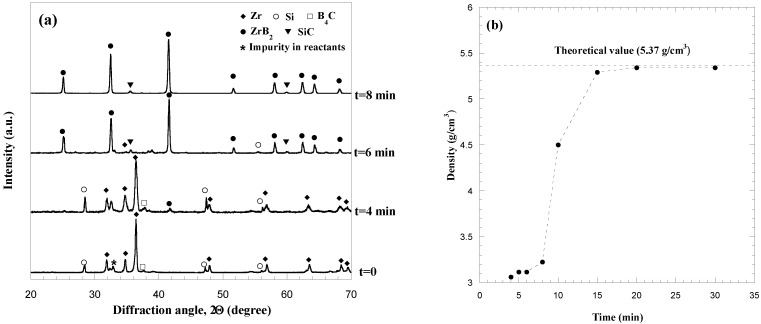
Compositional (**a**) and sample density (**b**) changes as a function of the sintering time during the reactive SPS of ZrB_2_–SiC (*T*_D_ = 1900 °C, *P* = 20 MPa). Adapted from [19].

Let us first examine the preparation of bulk ZrB_2_–SiC using the R-SPS method according to Reaction (2) with M = Zr. During the process, the temperature was increased from the room value to 1900 °C (*T*_D_) in 10 min and a constant applied pressure equal to 20 MPa was set. In contrast to the findings resulting when investigating the monolithic systems, the study of the formation mechanism of this composite material evidenced that the synthesis of ZrB_2_ and SiC starting from the Zr, B_4_C and Si does not occur under combustion regime but it proceeds gradually. In particular, as shown in Figure 6a, the first evidence of new phases formation (ZrB_2_) is found at 4 min while the compact undergoing sintering is still rich of reactants. The latter ones are almost completely converted within 6 min and a product containing only the desired phases is obtained after 8 min. Since the temperature levels measured when *t*_T_ ≤ 8 min were below 1200 °C, so that all starting reactants and final products are still solid, the formation of both the composite phases by R-SPS is likely governed by a solid-state diffusion mechanism. 

The evolution of the corresponding densification phenomena can be deduced from Figure 6b. It is seen that, despite of the complete reactants conversion, samples obtained when *t*_T_ = 8 min are still extremely porous. However, as the processing time is raised from 8 to 15 min, the densification level markedly improved from 60% to 98.5%. Moreover, a further increase of the synthesis time to 20 min leads to nearly full dense products (>99.5%). These findings are important as they provide the evidence that the conversion of starting reactants to the desired phases only modestly contributes to the densification, since higher densities are achieved only through the sintering of the composite products. This is another, albeit indirect, evidence of the occurrence of the synthesis reaction between solid state phases.

The fabrication of this UHTC composite product by SPS was also carried out using SHS powders as starting material. In this regard, according to the high enthalpy of Reaction (2) when M = Zr ($-\Delta H_{r}^{o}$ = 647.266 kJ) [60], a self-propagating front with a velocity equal to 11 mm/s was established in the reacting mixture upon ignition. Correspondingly, the maximum combustion temperature was about 2200 °C. In addition, XRD analysis showed that the synthesis reaction went to completion with the formation of the desired diboride and carbide phases with no secondary phases.

The obtained SHS product was then processed in the SPS apparatus after being ground to achieve particle size less than 20 μm. 

**Figure 7:**
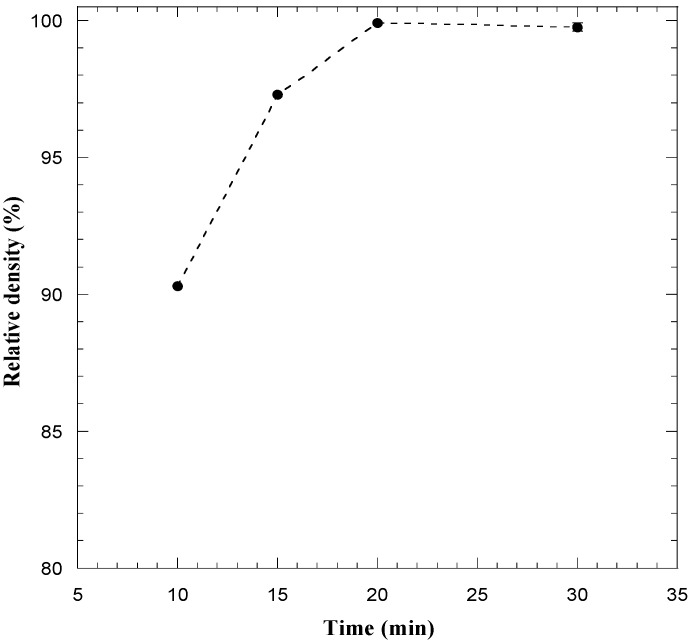
Influence of the sintering time on sample density during consolidation by SPS (*T*_D_ = 1800 °C, *P* = 20 MPa) of ZrB_2_–SiC powders obtained by SHS. Adapted from [19].

The dependence of the SPS time on product density was investigated when setting the *T*_D_ value and the applied pressure equal to 1800 °C and 20 MPa, respectively, and the obtained results are plotted in Figure 7. The residual porosity present in the material at the end of the non-isothermal step (10 min), is markedly reduced when holding the sample at the dwell temperature for 5 min, and, finally, a fully dense material (>99.5%) was produced in 20 min total time. 

From the comparison of the two processing routes utilized for the fabrication of fully dense ZrB_2_–SiC, it is clear that relatively milder sintering conditions (*T*_D_ = 1800 °C, *P* = 20 MPa, *t*_T_ = 20 min) are required when the SPS apparatus was used for the consolidation of SHS powders with respect to the case when the reactive sintering approach (*T*_D_ = 1900 °C, *P* = 20 MPa, *t*_T_ = 30 min) was adopted. 

This outcome is apparently in contrast with the experimental observations previously described when taking into account the fabrication of single-phase diboride materials. Nevertheless, this behavior can be justified as follows. First of all, different mechanisms govern the reactive SPS processes when considering the monolithic or composite systems addressed in this work. Specifically, a combustion synthesis regime is established only during the sintering of HfB_2_ and TaB_2_ from elemental reactants. Thus, the contribution to powders consolidation provided by the rapid sample shrinkage observed in Figure 2a is possible only when processing such systems. In contrast, the synthesis of ZrB_2_–SiC during R-SPS occurs gradually through a solid-state diffusion mechanism. 

Another possible complementary explanation can be provided based on considerations reported in the introductory section, relative to the important role played by the highly sintering character of SHS composite powders. Specifically, when the different ceramic phases are simultaneously formed *in situ* during SHS, stronger bonds are established at their interfaces. Consequently, diffusion distances in the composite powders are correspondingly reduced so that sintering phenomena are promoted.

The obtained results prompted us to follow the two steps processing route (SHS and SPS) also for the preparation of the HfB_2_–SiC and TaB_2_–SiC composites, which will be considered next.

#### 3.2.2. HfB_2_–SiC

Similarly to the systems examined previously, the exothermicity of Reaction (2) with M = Hf is responsible for the self-propagating character exhibited by the synthesis reaction. However, the enthalpy of reaction ($-\Delta H_{r}^{o}$ = 411.287 kJ) [60] is in this case lower in comparison to that relative to the formation of 2ZrB_2_–SiC. This fact is consistent with the relatively lower combustion temperature and average front velocity, equal to 2150 °C and 7 mm/s, respectively, correspondingly measured. The obtainment of the complete reactants conversion after SHS is confirmed by XRD analysis, where no undesired phases were revealed in the end-product.

**Figure 8:**
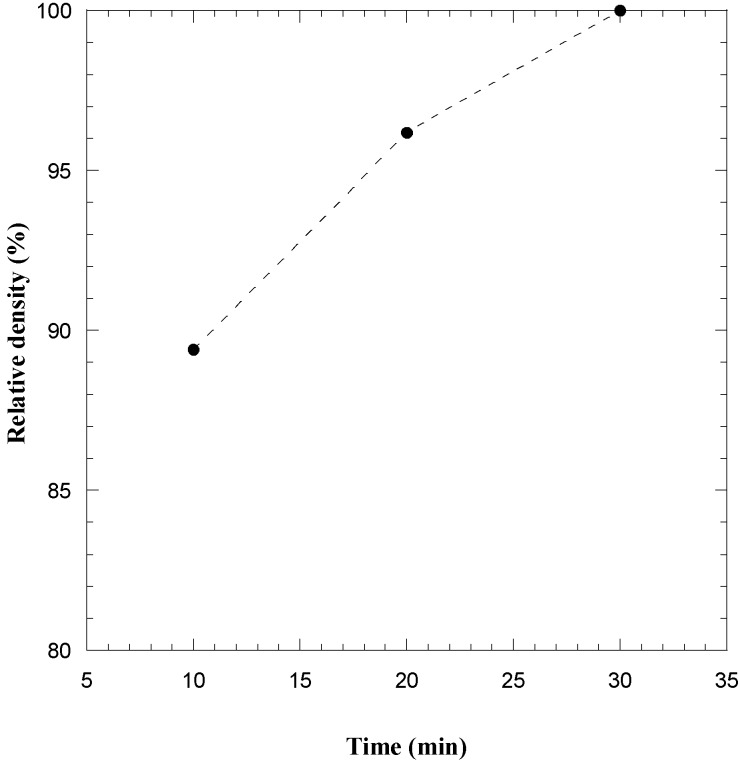
Influence of the sintering time on sample density during consolidation by SPS (*T*_D_ = 1800 °C, *P* = 20 MPa) of HfB_2_–SiC powders obtained by SHS. Adapted from [48].

The dependence of product density on the SPS time during the consolidation of HfB_2_–SiC is shown in Figure 8 for the case when the dwell temperature and the applied pressure were set to 1800 °C and 20 MPa, respectively. It is seen that the bulk material obtained after 20 min was not fully consolidated, in contrast to the case of the ZrB_2_–SiC system (*cf.* Figure 7). This difference can be readily ascribed to the higher melting point of HfB_2_ (3380 °C) as compared to ZrB_2_ (3245 °C). In any case, if the sintering time is prolonged of 10 additional min, the complete densification of the 2HfB_2_–SiC composite is achieved, thus confirming the efficacy of the adopted processing route.

#### 3.2.3. TaB_2_–SiC

In contrast to the reactive behavior displayed by the Zr- and Hf- binary systems, the attempts carried out to make self-sustaining the synthesis Reaction (2) with M = Ta failed when starting from as received powders. This outcome can be readily ascribed to the relatively lower enthalpy of reaction ($-\Delta H_{r}^{o}$ *=* 348.364 kJ) [60] with respect to the analogous systems taken into account in this work. Therefore, starting mixtures have received a suitable high energy ball milling (BM) treatment to activate initial reactants, thus successfully promoting the self-propagating character during the synthesis process. Correspondingly, the measured combustion temperature and front velocity were equal to 1850 ± 50 °C and 4.5 ± 0.5 mm/s, respectively. These values provide a further indication of the relatively low exothermic character of Reaction (2) with M = Ta.

The need of reactants activation in Ta-based ceramic composites is consistent with the behavior also observed during the preparation by SHS of TaB_2_–TaC from Ta, B_4_C and graphite powders [61]. Specifically, the propagation of the reaction front was made possible after preheating the initial mixture at 200 °C.

**Figure 9:**
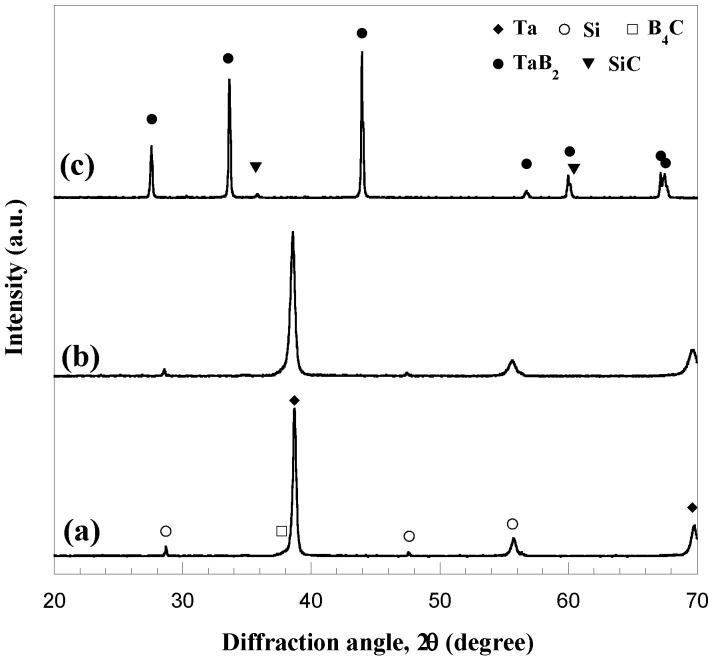
XRD analysis results during the synthesis of TaB_2_–SiC composite by SHS: (**a**) original reactants; (**b**) ball milled reactants and (**c**) products. Adapted from [51].

The composition of the SHS product obtained in the present study is reported in Figure 9 along with that of the starting reactants. Other than a slight peaks broadening as a manifestation of crystal size refinement and internal strain increase in the processing powders, no additional marked changes are evidenced by XRD analysis after the BM treatment. Nevertheless, these features and, above all, the interfaces formation among reactants, which allows for overcoming the diffusion limitations present when using unmilled powders, certainly contributes to the increased chemical reactivity. Figure 9 also shows that the mechanical activation received by reactants is sufficient to produce by SHS porous materials containing only the two desired composite constituents. 

**Figure 10:**
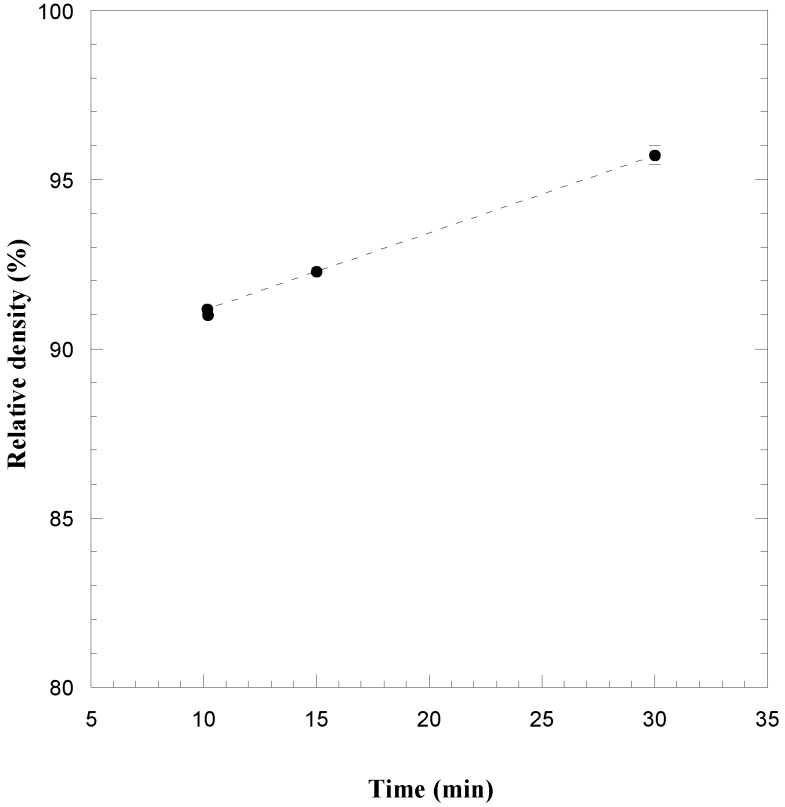
Influence of the sintering time on sample density during consolidation by SPS (*T*_D_ = 1800 °C, *P* = 20 MPa) of TaB_2_–SiC powders obtained by mechanically activated SHS. Adapted from [51].

The effect of the sintering time during the consolidation by SPS of the SHS powders was investigated by setting *T*_D_ = 1800 °C, being this thermal level achieved after heating the sample from the ambient temperature in 10 min, and *P* = 20 MPa. The obtained results are shown in Figure 10. It is seen that product density increases from about 91% of the theoretical value (at the end of the non-isothermal heating stage) to about 96%, when the sample was maintained at the dwell temperature for 20 more min. Therefore, highly dense materials can be also obtained for the case of the TaB_2_–SiC system.

## 4. Conclusions

The fabrication of dense monolithic (HfB_2_ and TaB_2_) and composite (ZrB_2_/SiC, HfB_2_/SiC, TaB_2_/SiC, with molar ratio equal to 2) UHTC materials was investigated taking advantage of Spark Plasma Sintering technology. Specifically, the results obtained using the following two processing routes were compared:

(a) Reactive sintering, where the *in situ* synthesis and consolidation of the material was performed in one processing step starting from appropriate reaction promoters
(b) Classical sintering, where the ceramic powders to be sintered by SPS were first synthesized by SHS using the same reactants as stated in (a)

Under the conditions experimentally investigated in this work, it was found that the kinetic mechanisms governing the reactions for the syntheses (approach (a)) of the monolithic and composite systems were different, *i.e.*, combustion-type and gradual solid-state diffusion, respectively. Moreover, when the combustion reaction takes place, a beneficial effect is obtained if a two step mechanical pressure cycle is implemented during the R-SPS process, *i.e.*, the applied load is increased immediately after the synthesis occurrence.

Furthermore, the results also indicate that method (a) is preferable to (b) for the preparation of highly dense monolithic systems. In contrast, the opposite outcome is found when considering the binary composite, where relatively milder sintering conditions are required to obtain fully dense materials when starting from SHS powders. 

The possible explanation of such behavior is related to the different mechanisms of reaction involved during R-SPS when the monolithic or composite systems were taken into account. Indeed, the sharp sample displacement taking place during the combustion synthesis event (monolithic systems) apparently promotes product densification. On the other hand, the solid diffusion mechanism governing the formation of the UHTC composite is accompanied by a gradual and slower sample consolidation. 

However, regardless of the monolithic or composite nature of the systems, although the relatively higher defect concentration and fine grains generated in the obtained product by the extreme heating and cooling conditions encountered during SHS process evolution are responsible for their good sintering ability, an additional positive contribution is provided for the case of materials containing two or more phases. Specifically, if the different ceramic constituents are simultaneously formed by SHS, stronger bonds can be established at their interfaces during the corresponding *in situ* synthesis. Thus, diffusion resistances are consequently reduced, so that sintering phenomena are favored. 

In general, it is important to emphasize the beneficial effects produced in both routes (a) and (b) by the use of the SPS technology. In this regard, the direct passage of the electric pulsed current through the sintering powders and the die containing them leads to very high heating rates, so that processing times can be significantly shortened and, usually, sintering temperatures lowered, with respect to traditional HP.
